# Sarcoidosis is associated with lower risks of penetrating disease and colectomy in hospitalized patients with inflammatory bowel disease

**DOI:** 10.1002/jgh3.12423

**Published:** 2020-10-05

**Authors:** Yi Jiang, Daniel S Rim, Brandon Rodgers, Sushil Ahlawat

**Affiliations:** ^1^ Department of Medicine Rutgers New Jersey Medical School Newark New Jersey USA; ^2^ Division of Gastroenterology and Hepatology Rutgers New Jersey Medical School Newark New Jersey USA

**Keywords:** colectomy, inflammatory bowel disease, penetrating disease, sarcoidosis

## Abstract

**Background and Aim:**

Inflammatory bowel disease (IBD) and sarcoidosis, primarily considered distinct entities, share commonalties in pathophysiology and clinical manifestations. This study aimed to examine the in‐hospital outcomes of patients with concurrent IBD and sarcoidosis.

**Methods:**

The National Inpatient Sample was used to identify hospitalized adult patients with IBD and sarcoidosis from 2010 to 2014. Primary outcomes were in‐hospital mortality, rates of septic shock, acute renal failure, respiratory failure, length of stay, and total hospitalization charges. Secondary outcomes were IBD‐specific complications and surgery interventions.

**Results:**

A total of 3995 patients with IBD and coexisting sarcoidosis (IBD/sarcoidosis), of which 2500 patients had Crohn's disease with coexisting sarcoidosis (Crohn's disease [CD]/sarcoidosis) and 1495 patients had ulcerative colitis with coexisting sarcoidosis (ulcerative colitis [UC]/sarcoidosis), were included. Patients with IBD/sarcoidosis had a lower risk of penetrating disease (adjusted odds ratio [aOR] 0.3, 95% confidence interval [CI] 0.16–0.55, *P* < 0.0001) and colectomy (aOR 0.48, 95% CI 0.27–0.84, *P* < 0.05). Subgroup analysis demonstrated lower rates of colectomy when comparing CD/sarcoidosis (*P* < 0.05) and UC/sarcoidosis (*P* = 0.0003) *versus* CD or UC alone. There was no difference in mortality.

**Conclusion:**

IBD/sarcoidosis is associated with lower risks of penetrating disease and colectomy when compared to patients with IBD alone.

## Graphical abstract

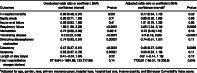

Sarcoidosis is associated with a lower rate of penetrating disease, leading to less colectomy in patients hospitalized with Crohn's disease and a higher rate of respiratory failure but a lower rate of colectomy in patients hospitalized with ulcerative colitis.

## Introduction

Inflammatory bowel disease (IBD), comprising ulcerative colitis (UC) and Crohn's disease (CD), and sarcoidosis are multifactorial disorders resulting from a complex interplay between genetic predisposition and environmental influence.^1^, ^2^, ^3^, ^4^ Although usually considered to be distinct entities, they share similar clinical and histopathological presentations. The underlying pathological changes in each disease result in the formation of granulomas^5^, ^6^ with resulting clinical overlap of ocular, dermatologic, and joint manifestations.^7^, ^8^, ^9^ Furthermore, patients with IBD can present with pulmonary disease,^5^, ^6^ and similar inflammatory changes that occur in the bowel and airway have been reported.^10^ Similarly, clinically recognizable gastrointestinal involvement has been observed in patients with sarcoidosis.^11^, ^12^

In fact, increasing amounts of immunological, bacteriological, and genetic data have supported a link between IBD and sarcoidosis.^13^ Observational studies first demonstrated nearly 50% positivity of Kveim testing in CD patients in the 1970s,^14^ which suggested a shared mechanism between CD and sarcoidosis. Since that initial study, a large amount of evidence has emerged, such as elevations of CD4/CD8 lymphocyte ratios in bronchoalveolar lavage fluid seen in both CD and sarcoidosis,^15^, ^16^, ^17^ as well as similar signs of response to mycobacterial infection in CD^18^ and sarcoidosis tissues.^19^ Concurrence of sarcoidosis and UC has also been previously reported. In addition, their pathogenic and immunogenic associations were suggested by several studies.^20^, ^21^, ^22^, ^23^ A nationwide Danish study also found a significantly increased risk of sarcoidosis in the population of patients with a diagnosis of UC or CD.^24^ Genome‐wide association studies have also found that IBD and sarcoidosis share susceptibility loci.^25^, ^26^, ^27^

Despite the tremendous progress in our understanding of the link or potential overlap of IBD and sarcoidosis, little is known regarding their clinical correlation. To date, there have been no large nationally representative studies to clarify the clinical outcomes in patients with coexisting IBD and sarcoidosis. In this nationwide study, we aimed to examine the outcomes (including length of stay [LOS], total hospitalization charges, in‐hospital mortality, and complications) for patients admitted with IBD who had coexisting sarcoidosis. Specifically, we investigated the disease‐/hospitalization‐related complications and their interventions, which can represent IBD severity^28^, ^29^, ^30^ in this patient population.

## Methods

### *Data source*

We obtained data from the Nationwide Inpatient Sample (NIS) database, which is the largest all‐payer inpatient care database in the United States. It is designed to approximate a 20% sample of the U.S. community hospitals. Yearly sampling weights are applied to generate national estimates.^31^ This database has been used previously to provide reliable estimate burdens of IBD^32^ and sarcoidosis.^33^ The data include demographic information (age, gender, and race); hospital information (bed size and teaching status); insurance status; discharge disposition; diagnoses and procedures identified by The International Classification of Diseases‐Ninth Edition Revision, Clinical Modification (ICD‐9 CM) codes; total hospital charges; and LOS.

### *Study population*

Our study was a retrospective cohort study of adult (18–90 years old) patients hospitalized with coexisting diagnosis codes for IBD (either CD or UC, ICD‐9 CM codes: 555.x and 556.x, respectively) and sarcoidosis (ICD‐9 CM code: 135) in the United States from 2010 to 2014. The control group was all IBD patients without a diagnosis of sarcoidosis (IBD alone group). Subgroup analysis was performed to compare coexisting CD and sarcoidosis *versus* CD alone, as well as coexisting UC and sarcoidosis *versus* UC alone. Demographic and clinical characteristics were collected (Table 1). Elixhauser Comorbidity Index^34^ (ECI), which measures 29 common medical conditions and assigns different weights to compile a score, was used to analyze the severity of comorbidities. Procedures and interventions during hospitalization, such as mechanical ventilation (MV), prolonged MV (more than 96 h MV), total parental nutrition (TPN), and central venous catheterization (CVC), were also included. All ICD‐9 CM codes are provided in Table S1, Supporting information.

**Table 1:** Comparison of selected variables of patients hospitalized with inflammatory bowel disease (IBD) with and without coexisting sarcoidosis

Variable	IBD with sarcoidosis	IBD without sarcoidosis	*P*‐value
N (weighted)	*n* = 3995	*n* = 3995	
Age	54.5 ± 0.6	55.4 ± 0.2	0.29
ECI score	5 ± 0.4	5.1 ± 0.1	0.88
Length of stay (days)	5.9 ± 0.3	5.5 ± 0.2	0.25
Total hospitalization charges (dollars)	48 338.3 ± 3204.2	42 621.7 ± 2529	0.15
Female	2590 (64.8)	2490 (62.3)	0.34
Caucasian	2420 (60.6)	2465 (61.7)	0.47
Large hospital bed	2535 (63.5)	2635 (66)	0.54
Urban teaching hospital	2350 (58.8)	2500 (62.6)	0.058
Medicare	1765 (44.2)	1910 (47.8)	0.68
Routine disposition	2730 (68.4)	2690 (67.3)	0.68
Mechanical ventilation	235 (5.9)	180 (4.5)	0.23
Prolonged mechanical ventilation	40 (1)	65 (1.6)	0.24
Total parenteral nutrition	135 (3.4)	150 (3.8)	0.69
Central venous catheterization	165 (4.1)	165 (4.1)	1.00
In‐hospital mortality	55 (1.4)	80 (2)	0.34
Septic shock	95 (2.4)	100 (2.5)	0.86
Acute renal failure	530 (13.3)	555 (13.9)	0.69
Respiratory failure	325 (8.1)	180 (4.5)	0.0033
Malnutrition	165 (4.1)	225 (5.6)	0.15
Penetrating disease	70 (1.8)	225 (5.6)	<0.0001
Stricturing disease/bowel obstruction	265 (6.6)	350 (8.8)	0.09
Colectomy	95 (2.4)	195 (4.9)	0.0067
Ileostomy	50 (1.3)	95 (2.4)	0.09

Values were reported as weighted means ± standard errors and weighted numbers (%).

ECI, Elixhauser Comorbidity Index.

The primary outcomes were in‐hospital outcomes, including mortality, septic shock, acute renal failure, respiratory failure, LOS, and total hospitalization charges. Our secondary outcomes included the IBD‐specific outcomes, including known complications of IBD such as malnutrition, penetrating disease, stricturing disease/bowel obstruction, and surgical interventions such as colectomy and ileostomy.

### *Statistics*

SAS Survey Procedures (SAS 9.4, SAS Institute Inc., Cary, NC, USA) was used for all statistical analyses. The propensity scoring method^35^, ^36^ was used to select the control group (IBD patients without coexisting sarcoidosis) in order to mitigate selection bias and to control for patient and institutional imbalances. The scoring was based on a multivariate logistic regression model accounting for age, gender, race, primary insurance payer, hospital type, hospital bed size, hospital region, and hospital teaching status. Using an 8‐to‐1‐digit match, we paired each admission in the IBD coexisting sarcoidosis group with one admission in the IBD without sarcoidosis group.

The national estimates were calculated after accounting for the sample design elements (clusters, strata, and trend weights) provided by the NIS. Continuous variables were reported as weighted means ± standard errors (SEs), and categorical variables were reported as weighted numbers (N) and percentages (%). The SEs of weighted means were estimated by using the Taylor linearization method that incorporates the sample design. The Rao‐Scott‐modified chi‐square test was used to test the difference in distribution for categorical variables, while a weighted Student's *t*‐test was used to analyze the normally distributed continuous variables. Variables that are not normally distributed were tested by the Wilcoxon rank‐sum tests.

A multivariate logistic regression was used to estimate the odds ratio of in‐hospital mortality, septic shock, acute renal failure, respiratory failure, IBD complications, and surgical interventions. Confounders included in the regression analysis were patient demographics, hospital bed size, hospital location/teaching status, insurance type, median household income, and ECI score. In addition, a multivariate linear regression was used to estimate the average change in LOS and total hospital charges after adjusting for the same covariates described above.

## Results

### *Patient characteristics*

In this study, a total of 302 105 patients were admitted for IBD, of which 192 290 patients had CD and 109 815 patients had UC. A total of 3995 IBD patients were diagnosed with coexisting sarcoidosis (IBD/sarcoidosis), of which 2500 patients had CD coexisting with sarcoidosis (CD/sarcoidosis) and 1495 patients had UC coexisting with sarcoidosis (UC/sarcoidosis). After matching, the IBD/sarcoidosis, CD/sarcoidosis, and UC/sarcoidosis groups were compared to equal numbers of patients with IBD, CD, and UC without coexisting sarcoidosis, respectively. IBD/sarcoidosis patients were an average of 54.5 years old, of which 64.8% were female, 60.6% were Caucasian, and the patients were mostly admitted to large (63.5%) and urban teaching (58.8%) hospitals with an average ECI score of 5. No statistical difference was found between the IBD/sarcoidosis and IBD alone groups in terms of patient demographics (age, gender, race), hospital characteristics (bed size and teaching status), and ECI score (Table 1). In the IBD/sarcoidosis group, 5.9% of patients received MV, 1% had prolonged MV, 3.4% patients received TPN, and 4.1% had CVC during hospitalization. Again, no difference was found for the above interventions when compared to IBD alone group.

### *In‐hospital outcomes and multivariate analysis*

Interestingly, our analysis revealed lower rates of penetrating disease (1.8 *vs* 5.6%, *P* < 0.001) and colectomy (2.4 *vs* 4.9%, *P* < 0.05) in the IBD/sarcoidosis group compared with the IBD alone group. Not surprisingly, IBD/sarcoidosis was associated with a higher rate of respiratory failure (8.1 *vs* 4.5%, *P* < 0.05). Although we observed lower rates of hospital mortality (1.4 *vs* 2%, *P* = 0.34), malnutrition (4.1 *vs* 5.6%, *P* = 0.15), stricturing disease/bowel obstruction (6.6 *vs* 8.8%, *P* = 0.09), and ileostomy (1.3 *vs* 2.4%, *P* = 0.09) in the IBD/sarcoidosis patient cohort, these results were not statistically significant. In addition, there was no difference in the rates of septic shock, acute renal failure, and hospitalization burden in terms of LOS or total hospitalization charges found between the IBD/sarcoidosis and IBD alone groups. Upon multivariate analysis (Table 2), patients with IBD/sarcoidosis were observed to have a significantly higher rate of respiratory failure (adjusted odds ratio [aOR] 2.05, 95% confidence interval [CI] 1.34–3.14, *P* < 0.001) compared to patients with IBD alone. In terms of IBD‐specific complications, IBD/sarcoidosis was independently associated with a significantly lower rate of penetrating disease (aOR 0.3, 95% CI 0.16–0.55, *P* < 0.0001) compared to IBD alone. In addition, IBD/sarcoidosis was also associated with a lower rate of colectomy (aOR 0.48, 95% CI 0.27–0.84, *P* < 0.05) when compared to IBD alone. Interestingly, after adjusting for confounders, we found higher total hospitalization charges (adjusted coefficient 7730.91, 95% CI 155.31–15 306.5, *P* < 0.05) in the IBD/sarcoidosis group when compared to the IBD alone group.

**Table 2:** Multivariate regression analysis of outcomes for hospitalized patients with inflammatory bowel disease with and without coexisting sarcoidosis

Outcomes	Unadjusted odds ratio or coefficient (95% confidence interval)	*P*‐value	Adjusted odds ratio or coefficient (95% confidence interval)^†^	*P*‐value
In‐hospital mortality	0.68 (0.48, 0.97)	0.031	0.77 (0.34, 1.73)	0.53
Septic shock	0.95 (0.71, 1.26)	0.71	0.99 (0.52, 1.86)	0.96
Acute renal failure	0.95 (0.83, 1.08)	0.41	1.01 (0.75, 1.35)	0.96
Respiratory failure	1.88 (1.56, 2.26)	<0.0001	2.05 (1.34, 3.14)	0.0009
Malnutrition	0.72 (0.59, 0.89)	0.0019	0.73 (0.46,1.16)	0.18
Penetrating disease	0.3 (0.23, 0.39)	<0.0001	0.3 (0.16, 0.55)	<0.0001
Stricturing disease/bowel obstruction	0.74 (0.63, 0.87)	0.00037	0.74 (0.51, 1.07)	0.11
Colectomy	0.47 (0.37, 0.61)	<0.0001	0.48 (0.27, 0.84)	0.0099
Ileostomy	0.52 (0.37, 0.73)	0.00021	0.53 (0.24, 1.16)	0.11
Length of stay (days)	0.4 (−0.24, 1.04)	0.22	0.51 (−0.14, 1.15)	0.12
Total hospitalization charges (dollars)	5716.64 (−1884.59, 133 317.88)	0.14	7730.91 (155.31, 15 306.5)	0.045

† Adjusted for age, gender, race, primary insurance payer, hospital type, hospital bed size, income quartile, and Elixhauser Comorbidity Index score.

### *Subgroup analysis*

Subsequent comparisons between CD/sarcoidosis and CD and UC/sarcoidosis and UC were performed. Compared to CD alone, CD/sarcoidosis had comparable demographic characteristics in terms of age, gender, and race (Table 3). No difference was found in hospital type, hospital teaching status, or ECI score between the CD/sarcoidosis and CD alone groups. Regarding interventions during hospitalization in the CD/sarcoidosis group, 4.4% patients received MV, 0.8% had prolonged MV, 3.4% patients received TPN, and 4.1% had CVC. There was no difference in intervention between the CD/sarcoidosis and the CD groups. In‐hospital outcomes analysis revealed lower rates of penetrating disease (2.8 *vs* 9%, *P* < 0.0001) and colectomy (1.6 *vs* 3.8%, *P* < 0.05) in the CD/sarcoidosis group compared to the CD alone group. After adjusting for confounders, this result remained significant for less penetrating disease (aOR 0.29, 95% CI 0.15–0.53, *P* < 0.0001) and colectomy (aOR 0.42, 95% CI 0.18–0.96, *P* < 0.05) in patients with CD/sarcoidosis. (Table 4).

**Table 3:** Comparison of selected variables of patients hospitalized with Crohn's disease (CD) with and without coexisting sarcoidosis

Variable	CD with sarcoidosis	CD without sarcoidosis	*P*‐value
N (weighted)	*n* = 2500	*n* = 2500	
Age	53.3 ± 0.8	54.9 ± 0.7	0.11
ECI score	4.1 ± 0.5	4.5 ± 0.4	0.50
Length of stay (days)	5.6 ± 0.3	5.2 ± 0.3	0.33
Total hospitalization charges (dollars)	42 376.5 ± 3350.4	36 729.4 ± 1919.1	0.14
Female	1725 (69)	1665 (62.3)	0.34
Caucasian	1450 (58)	1515 (60.6)	0.09
Large hospital bed	1520 (60.8)	1545 (61.8)	0.76
Urban teaching hospital	1445 (57.8)	1570 (62.8)	0.063
Medicare	1145 (45.8)	1225 (49)	0.81
Routine disposition	1765 (70.7)	1690 (67.6)	0.58
Mechanical ventilation	110 (4.4)	105 (4.2)	0.88
Prolonged mechanical ventilation	20 (0.8)	20 (0.8)	1.00
Total parenteral nutrition	85 (3.4)	105 (4.2)	0.53
Central venous catheterization	75 (3)	105 (4.2)	0.31
In‐hospital mortality	30 (1.2)	40 (1.6)	0.60
Septic shock	40 (1.6)	40 (1.6)	1.00
Acute renal failure	310 (12.4)	335 (13.4)	0.62
Respiratory failure	160 (6.4)	110 (4.4)	0.17
Malnutrition	90 (3.6)	150 (6)	0.059
Penetrating disease	70 (2.8)	225 (9)	<0.0001
Stricturing disease/bowel obstruction	265 (10.6)	350 (14)	0.08
Colectomy	40 (1.6)	95 (3.8)	0.0268
Ileostomy	10 (0.4)	10 (0.4)	1.00

Values were reported as weighted means ± standard errors and weighted numbers (%).

ECI, Elixhauser Comorbidity Index.

**Table 4:** Multivariate regression analysis of outcomes for hospitalized patients with Crohn's disease with and without coexisting sarcoidosis

Outcomes	Unadjusted odds ratio or coefficient (95% confidence interval)	*P*‐value	Adjusted odds ratio or coefficient (95% confidence interval)†	*P*‐value
In‐hospital mortality	0.75 (0.46, 1.21)	0.23	0.88 (0.29, 2.67)	0.82
Septic shock	1 (0.64, 1.56)	1.00	1.04 (0.39, 2.79)	0.93
Acute renal failure	0.91 (0.78, 1.08)	0.29	0.99 (0.67, 1.44)	0.94
Respiratory failure	1.49 (1.16, 1.91)	0.0019	1.66 (0.94, 2.93)	0.08
Malnutrition	0.59 (0.45, 0.76)	0.0001	0.59 (0.32,1.08)	0.09
Penetrating disease	0.29 (0.22, 0.38)	<0.0001	0.29 (0.15, 0.53)	<0.0001
Stricturing disease/bowel obstruction	0.73 (0.61, 0.86)	0.0003	0.73 (0.5, 1.07)	0.11
Colectomy	0.41 (0.28, 0.6)	<0.0001	0.42 (0.18, 0.96)	0.041
Ileostomy	1 (0.42, 2.41)	1.00	1.19 (0.18, 7.83)	0.85
Length of stay (days)	0.4 (−0.36, 1.17)	0.30	0.53 (−0.24, 1.3)	0.18
Total hospitalization charges (dollars)	5647.09 (−2014.33, 13 308.51)	0.15	7509.88 (−163.86, 15 183.62)	0.055

† Adjusted for age, gender, race, primary insurance payer, hospital type, hospital bed size, income quartile, and Elixhauser Comorbidity Index score.

There was no significant difference with regard to age, gender, race, hospital type, hospital teaching status, and ECI score between the UC/sarcoidosis and UC alone groups (Table 5). In the UC/sarcoidosis group, 8.4% patients received MV, of which 1.3% had prolonged MV. 3.3% had TPN, and 6% received CVC. These intervention rates were not significantly different between the UC/sarcoidosis and the UC alone groups. UC/sarcoidosis was associated with a significantly higher rate of respiratory failure (11 *vs* 4.7%, *P* = 0.0025) compared to UC alone. After adjusting for confounders (Table 6), UC/sarcoidosis continued to have a higher rate of respiratory failure (aOR 2.66, 95% CI 1.39–5.1, *P* = 0.0031) when compared to UC alone. Multivariate regression analysis also showed a lower rate of colectomy (aOR 0.53, 95% CI 0.38–0.75, *P* = 0.0003) in the UC/sarcoidosis group when compared to the UC alone group.

**Table 5:** Comparison of selected variables of patients hospitalized with ulcerative colitis (UC) with and without coexisting sarcoidosis

Variable	UC with sarcoidosis	UC without sarcoidosis	*P*‐value
N (weighted)	*n* = 1495	*n* = 1495	
Age	56.5 ± 0.8	56.1 ± 1	0.75
ECI score	6.5 ± 0.6	6.1 ± 0.5	0.59
Length of stay (days)	6.4 ± 0.5	6 ± 0.4	0.53
Total hospitalization charges (dollars)	58 220.7 ± 5878	62 428.6 ± 5180.5	0.51
Female	865 (57.9)	825 (55.2)	0.48
Caucasian	970 (64.9)	950 (63.5)	0.79
Large hospital bed	1015 (67.9)	1090 (72.9)	0.31
Urban teaching hospital	905 (60.5)	930 (62.2)	0.57
Medicare	620 (41.5)	685 (45.8)	0.72
Routine disposition	965 (64.5)	1000 (66.9)	0.31
Mechanical ventilation	125 (8.4)	75 (5)	0.081
Prolonged mechanical ventilation	20 (1.3)	45 (3)	0.10
Total parenteral nutrition	50 (3.3)	45 (3)	0.79
Central venous catheterization	90 (6)	60 (4)	0.25
In‐hospital mortality	25 (1.7)	40 (2.7)	0.35
Septic shock	55 (3.7)	60 (4)	0.82
Acute renal failure	220 (14.7)	220 (14.7)	1.00
Respiratory failure	165 (11)	70 (4.7)	0.0025
Malnutrition	75 (5)	75 (5)	1.00
Colectomy	55 (3.7)	100 (6.7)	0.10
Ileostomy	40 (2.7)	85 (5.7)	0.065

Values were reported as weighted means ± standard errors and weighted numbers (%).

ECI, Elixhauser Comorbidity Index.

**Table 6:** Multivariate regression analysis of outcomes for hospitalized patients with ulcerative colitis with and without coexisting sarcoidosis

Outcomes	Unadjusted odds ratio or coefficient (95% confidence interval)	*P*‐value	Adjusted odds ratio or coefficient (95% confidence interval)†	*P*‐value
In‐hospital mortality	0.62 (0.37, 1.02)	0.062	0.65 (0.2, 2.1)	0.47
Septic shock	0.91 (0.63, 1.33)	0.63	0.92 (0.4, 2.13)	0.85
Acute renal failure	1 (0.82, 1.22)	1.00	1.05 (0.65, 1.68)	0.85
Respiratory failure	2.53 (1.89, 3.37)	<0.0001	2.66 (1.39, 5.1)	0.0031
Malnutrition	1 (0.72, 1.39)	1.00	1 (0.47, 2.11)	1.00
Colectomy	0.53 (0.38, 0.75)	0.0003	0.53 (0.38, 0.75)	0.0003
Ileostomy	0.46 (0.31, 0.67)	<0.0001	0.44 (0.18, 1.04)	0.061
Length of stay (days)	0.39 (−0.76, 1.54)	0.50	0.44 (−0.71, 1.6)	0.45
Total hospitalization charges (dollars)	5792.13 (−9845.59, 21 429.85)	0.47	7356.44 (−8326.58, 23 039.46)	0.36

† Adjusted for age, gender, race, primary insurance payer, hospital type, hospital bed size, income quartile, and Elixhauser Comorbidity Index score.

## Discussion

This nationwide study examined the in‐hospital outcomes of patients with IBD/sarcoidosis compared to IBD alone. Subsequent subgroup analysis further compared CD/sarcoidosis *versus* CD alone and UC/sarcoidosis *versus* UC alone. The major findings were that IBD/sarcoidosis was associated with a significantly lower rate of penetrating disease, fewer colectomies, and a higher rate of respiratory failure compared to IBD alone. Subgroup analysis further specified that both CD/sarcoidosis and UC/sarcoidosis demonstrated lower risk of colectomy when compared to CD and UC alone, respectively. UC/sarcoidosis was particularly associated with a higher rate of respiratory failure.

The association of sarcoidosis with less penetrating disease and colectomy in IBD patients is incompletely understood and not previously reported. Here, we propose several hypotheses to explain this finding. First, this finding may suggest that the coexistence of sarcoidosis impacts disease subphenotype in IBD. It has been accepted that the location of IBD is clearly associated with different risks of complications.^37^ In CD, four subphenotypes have been classified based on location, with three exclusive subphenotypes (pure ileal disease, pure colonic disease, and ileocolonic disease) plus a fourth modifying subphenotype that may coexist with the three main subphenotypes: upper gastro‐intestinal tract location.^38^ Ileal involvement (pure ileal disease and ileoclonic disease), which comprises around 60% of CD at diagnosis,^39^, ^40^ has been associated with developing strictures and/or fistulas^40^, ^41^, ^42^ and therefore leads to the increased risk of surgery,^42^, ^43^ whereas left‐sided colonic CD is associated with a significant reduction in the risk of complications.^44^ In UC, the subphenotype at diagnosis is generally split equally between proctitis, left‐sided disease, and pancolitis.^45^ Extensive colitis in UC has been associated with an increased risk of colectomy.^46^ Taken together, it is reasonable to hypothesize that the coexistence of sarcoidosis in IBD is associated with less ileal involvement in CD and less pancolitis in UC and possibly more left‐sided localized disease. These dominant subphenotypes carry lower risks of penetrating disease and colectomy. In fact, it was well documented that, when IBD coexists with certain other diseases (i.e. primary sclerosing cholangitis), the phenotype can be completely shifted to nonstricture‐forming and nonpenetrating colonic disease.^47^, ^48^ Similarly, sarcoidosis may play a role in determining the subphenotypes of IBD as well.

The pathogenesis of ileal *versus* colonic CD has been extensively studied. ATG16L risk alleles, IL‐23/Th17 axis, αE+ T‐cells, and microbial community profiles in different key pathways have been shown to be significantly different between ileal and colonic CD.^49^ One important allele potentially related to sarcoidosis is the caspase recruitment domain‐containing protein 15 (CARD15) gene polymorphisms. CARD15 is associated with ileal CD.^50^ It has also been reported that early‐onset cases of sarcoidosis had heterozygous missense mutations in the CARD15 gene.^51^ Moreover, an association between two CARD15 polymorphisms and specific sarcoidosis phenotypes have been demonstrated.^52^ Whether sarcoidosis plays a role in the pathogenesis of a particular IBD subphenotype through the CARD15 pathway remains an intriguing possibility. No conclusion has been made thus far.

Second, aside from dominant location subphenotypes of IBD, the phenotype of endoscopic lesions (i.e. the size and depth of ulcers) has also likely been of important clinical significance^53^ in determining the rates of penetrating disease and subsequent surgery. Intestinal sarcoidosis is usually limited to the mucosa, whereas the inflammation in CD can be transmural.^54^ An additional hypothesis for the low rates of penetrating disease and colectomy in CD patients is that sarcoidosis may be associated with more superficial mucosal lesions when coexisting with CD. Further endoscopic investigation on the size and depth of mucosal lesions in IBD/sarcoidosis patients is needed.

In addition, a changing pattern of disease behavior has been described in IBD.^55^ On diagnosis of CD, most patients exhibit purely inflammatory behavior (nonstricturing, nonpenetrating). Ten years later, approximately one‐third of the patients were seen to have developed stricturing and another third fistulating disease.^56^ For patients with UC, approximately 50% of initial proctitis or left‐sided colitis spreads over time.^57^ It is likely that patients with coexisting IBD and sarcoidosis present with more clinically apparent symptoms and are more likely be admitted to the hospital at an early stage of IBD. Therefore, not many patients would have developed penetrating disease or experienced colectomy at the time of their admission.

As expected, our study revealed a high rate of respiratory failure in patients with IBD/sarcoidosis compared to the IBD alone group. It is well accepted that more than 90% of patients with sarcoidosis have evidence of intrathoracic involvement.^58^ It has also been reported that a patient's immunogenic background could underlie the heterogeneity of sarcoidosis and play a role in the clinical manifestation of the disease.^59^ The underlying lung disease may explain the high rate of respiratory failure during hospitalization. Although associated with higher rates of respiratory failure, there was no difference in MV or prolonged MV observed between the IBD/sarcoidosis and IBD only groups. This result may suggest that IBD is associated with a treatment‐sensitive phenotype of pulmonary sarcoidosis.

This study has several strengths. As a nationwide sample, the NIS database provided a large sample size to study the IBD/sarcoidosis population. Therefore, our results are based on a high‐power study and provide a national review of the disease. To the best of our knowledge, this is a leading study to evaluate the clinical outcomes of patients with coexisting IBD and sarcoidosis. Identification of significant clinical outcomes associated with the immunopathological and genetic association of IBD and sarcoidosis may aid in making decisions regarding diagnosis and treatment. Our study also has limitations. All diagnoses are dependent on the accuracy of ICD‐9 codes, for which validation is routinely performed by the Agency for Healthcare Research and Quality, although coding errors may compromise the quality of the data. This study's main diagnostic ICD‐9‐CM codes for IBD and sarcoidosis have been previously used and validated in different studies.^29^, ^30^, ^33^ An additional inherent limitation of the NIS database is that no laboratory values, images, or pharmacological interventions are recorded. Therefore, additional information that may help better identify disease activity and stage are not available. Future studies will be needed to address this information during the investigation of both IBD and sarcoidosis that are featured by a dynamic and heterogenic clinical picture.

In conclusion, this national study used a large dataset from the United States to examine the in‐hospital outcomes of IBD/sarcoidosis. Our results first identified that sarcoidosis is associated with unique clinical features when coexisting with IBD, including lower risk of penetrating disease and colectomy compared to IBD alone. Furthermore, we strengthened the previous understanding of pulmonary sarcoidosis, leading to significant respiratory complications for hospitalized patients. These results may suggest a specific clinical and pathological subphenotype of IBD when coexisting with sarcoidosis. Prospective studies at the genetic and clinicopathological levels will assist in gaining a better understanding of the mechanism of the interaction between IBD and sarcoidosis. Ultimately, more diagnostic and therapeutic strategies are expected.

## Supporting information


**Table S1** ICD‐9‐CM codes.
