# Supplementary material for: Sarcoidosis is associated with lower risks of penetrating disease and colectomy in hospitalized patients with inflammatory bowel disease
Source: JGH Open. 2020 Oct 5;4(6):1199–206. doi: 10.1002/jgh3.12423 (PMC7731821; doi:10.1002/jgh3.12423)
Supplement: Supplementary file 1 — Table S1 ICD‐9‐CM codes. [file JGH3-4-1199-s001.docx]

Supplementary Table 1. ICD-9-CM codes

| Variable/outcome | ICD-9 codes |
| --- | --- |
| Crohn’s disease | 555.0, 555.1, 555.2, and 555.9 |
| Ulcerative colitis | 556.0, 556.1-556.6, 556.8, and 556.9 |
| Sarcoidosis | 135.0 |
| Mechanical ventilation | 939.0, 939.2, 960.1-960.5, and 967.0-967.2 |
| Prolonged mechanical ventilation | 967.2 |
| Total parenteral nutrition | 991.5 |
| Central venous catheterization | 389.75 |
| Septic shock | 785.52 |
| Acute renal failure | 584.5-584.9 and 586.0 |
| Respiratory failure | 518.81, 518.82, 518.84, and 799.1 |
| Penetrating disease | 537.4, 567.21, 567.22, 569.5, 569.81-569.83, 596.1, and 619.1 |
| Stricturing disease/bowel obstruction | 560.0-560.2, 560.30, 560.39, 560.81, 560.89, 560.9, 537.3 |
| Malnutrition | 263.0-263.2, 263.8, 263.9 |
